# Supplementary material for: The Monetary Value of Human Lives Lost to Suicide in the African Continent: Beating the African War Drums
Source: Healthcare (Basel). 2020 Apr 2;8(2):84. doi: 10.3390/healthcare8020084 (PMC7348721; doi:10.3390/healthcare8020084)
Supplement: Supplementary file 1 [file healthcare-08-00084-s001.pdf]

healthcare MDPI

Table S1: Distribution of suicide deaths by age group (2017).

	Age groups																	
**Country**	10 to 14	15 to 19	20 to 24	25 to 29	30 to 34	35 to 39	40 to 44	45 to 49	50 to 54	55 to 59	60 to 64	65 to 69	70 to 74	75 to 79	80 to 84	85 to 89	90 to 94	95 plus
Algeria	23	128	229	243	243	204	152	110	85	62	54	41	28	26	31	24	10	4
Angola	41	101	155	148	131	137	141	147	167	151	125	108	82	62	32	13	3	1
Benin	12	38	59	60	54	56	49	57	55	51	45	45	41	32	23	10	3	1
Botswana	0	15	21	22	24	23	20	16	14	12	8	7	6	5	3	2	1	0
Burkina Faso	31	82	116	119	114	123	111	123	123	120	106	107	95	68	44	20	6	1
Burundi	19	40	59	59	52	53	52	53	62	62	56	48	36	26	15	6	1	0
Cameroon	41	123	186	196	180	183	156	171	170	167	147	144	126	90	61	26	6	1
Cape Verde	0	2	4	9	10	13	9	10	7	6	3	2	2	2	3	3	1	0
Central African Republic	10	31	47	43	40	46	50	55	56	46	35	27	18	10	5	1	0	0
Chad	21	50	68	63	55	59	53	62	63	59	53	55	54	36	26	11	3	1
Comoros	1	2	3	3	3	3	3	4	4	3	3	3	3	3	2	1	0	0
Congo	7	22	34	35	35	41	45	46	46	39	31	27	22	16	10	4	1	0
Cote d'Ivoire	41	96	147	161	159	173	152	162	158	153	130	127	107	72	50	22	5	1
Democratic Republic of the Congo	110	299	500	472	435	426	430	483	524	472	394	315	237	170	105	45	12	2
Equatorial	2	5	8	7	6	5	4	5	5	4	4	3	3	3	2	1	0	0

Guinea																		
Eritrea	13	34	57	57	51	55	56	53	49	39	30	28	18	12	6	2	0	0
Ethiopia	183	338	488	437	351	368	347	337	353	330	321	343	328	276	193	87	23	4
Gabon	2	7	11	11	11	11	12	14	16	16	14	11	9	7	5	2	1	0
The Gambia	5	2	0	0	2	7	11	11	9	9	8	9	9	8	8	8	8	7
Ghana	30	84	143	151	147	163	150	164	153	145	140	145	118	82	60	24	6	1
Guinea	13	39	60	59	53	53	47	55	57	59	56	61	52	37	27	14	4	1
Guinea-Bissau	2	8	13	15	14	13	11	12	11	10	9	8	7	4	2	1	0	0
Kenya	69	161	248	245	223	235	236	236	258	251	217	218	190	134	84	39	11	3
Lesotho	1	34	50	55	65	61	51	41	37	31	21	21	17	13	8	4	1	0
Liberia	5	16	25	26	26	29	28	31	28	26	23	23	21	14	11	6	2	0
Madagascar	41	91	148	142	120	128	130	136	150	139	108	91	69	49	30	14	4	1
Malawi	33	69	110	102	84	90	87	84	81	75	71	81	81	69	46	21	5	1
Mali	20	46	63	59	51	56	48	56	56	54	48	52	46	32	24	11	3	0
Mauritania	3	8	11	11	10	10	9	11	12	13	12	13	12	9	7	4	1	0
Mauritius	1	8	15	14	13	13	11	9	10	7	5	4	3	2	1	1	0	0
Mozambique	79	182	271	249	220	233	220	219	239	202	152	143	128	99	56	22	6	1
Namibia	0	20	27	25	24	23	22	20	18	14	9	8	7	5	4	2	1	0
Niger	18	48	69	65	57	61	48	54	61	64	53	53	49	27	16	8	2	0
Nigeria	240	482	660	630	553	572	507	607	610	606	516	509	484	421	357	217	77	21
South Sudan	20	34	46	40	39	48	52	55	57	49	40	35	32	24	16	8	2	0

Rwanda	19	41	59	60	54	55	50	52	64	69	64	59	48	39	27	13	3	1
Sao Tome and Principe	0	0	1	1	0	1	0	0	0	0	0	0	0	0	0	0	0	0
Senegal	20	55	79	76	65	69	65	77	90	99	90	95	87	65	49	24	7	1
Seychelles	0	0	1	1	1	1	1	1	1	1	0	0	0	0	0	0	0	0
Sierra Leone	8	27	42	43	37	37	31	34	32	31	28	29	29	22	15	6	2	0
South Africa	4	182	664	900	1009	746	543	486	459	395	255	187	127	92	75	58	28	10
Swaziland	0	17	27	27	29	26	22	17	13	10	6	5	4	2	1	0	0	0
Tanzania	80	136	193	187	164	185	185	201	220	204	182	185	174	155	111	55	17	4
Togo	8	26	44	46	45	50	48	55	54	48	38	39	30	19	12	5	1	0
Uganda	61	134	182	161	127	126	129	141	157	150	136	142	138	123	79	33	9	3
Zambia	33	66	108	100	84	91	97	95	95	83	69	69	64	53	34	15	4	1
Zimbabwe	8	236	285	259	283	285	259	226	177	157	131	120	96	64	36	17	6	2
Djibouti	1	3	4	5	4	5	5	6	6	5	5	4	3	2	1	1	0	0
Egypt	63	437	615	647	637	567	441	338	277	216	184	139	84	50	34	18	6	2
Libya	5	41	73	73	93	78	73	50	29	18	14	9	8	6	5	4	2	1
Morocco	28	205	313	308	299	286	256	190	159	143	118	76	59	44	44	30	14	5
Somalia	26	51	73	66	63	78	81	62	58	64	69	67	47	28	14	5	1	0
Sudan	41	206	297	247	209	169	133	98	71	49	40	28	22	16	14	9	4	2
Tunisia	3	18	32	39	43	37	32	25	24	24	21	17	14	12	16	13	6	2

Source: Global Burden of Disease Collaborative Network [5].

Table S2: Both sexes Life expectancy at birth (years) in continental Africa.

Member State	Life expectancy
Algeria	76.4
Angola	62.6
Benin	61.1
Botswana	66.1
Burkina Faso	60.3
Burundi	60.1
Cameroon	58.1
Cabo Verde	73.2
Central African Republic	53
Chad	54.3
Comoros	63.9
Congo	64.3
Côte d'Ivoire	54.6
Democratic Republic of the Congo	60.5
Equatorial Guinea	59.5
Eritrea	65
Ethiopia	65.5
Gabon	66.4
Gambia	61.9
Ghana	63.4
Guinea	59.8
Guinea-Bissau	59.8
Kenya	66.7
Lesotho	52.9
Liberia	62.9
Madagascar	66.1
Malawi	64.2
Mali	58
Mauritania	63.9
Mauritius	74.8
Mozambique	60.1
Namibia	63.7
Niger	59.8
Nigeria	55.2
Rwanda	68
Sao Tome and Principe	68.7
Senegal	66.8
Seychelles	73.3
Sierra Leone	53.1
South Africa	63.6
Swaziland	57.7

Togo	60.6
Uganda	62.5
Tanzania	63.9
Zambia	62.3
Zimbabwe	61.4
South Sudan	58.6
Djibouti	63.8
Egypt	70.5
Libya	71.9
Morocco	76
Somalia	55.4
Sudan	65.1
Tunisia	76

Source: WHO [18].

Table 3. Gross domestic product per capita and current health expenditure per capita in purchasing power parity or international dollars (2019 Int$).

Country	Gross domestic product per capita (Int$)	Current health expenditure per capita (Int$)
Algeria	16,086	998
Angola	6,930	186
Benin	2,552	83
Botswana	18,583	931
Burkina Faso	2,103	116
Burundi	730	62
Cameroon	3,974	169
Cabo Verde	7,765	349
Central African Republic	750	30
Chad	2,506	95
Comoros	1,669	116
Congo	7,109	263
Côte d'Ivoire	4,443	163
Democratic Republic of the Congo	843	34
Equatorial Guinea	33,381	839
Eritrea	1,725	55
Ethiopia	2,557	70
Gabon	19,431	556
Gambia	2,883	74
Ghana	5,386	189
Guinea	2,404	108
Guinea-Bissau	2,047	98
Kenya	3,896	144
Lesotho	3,465	243
Liberia	1,381	133
Madagascar	1,703	90
Malawi	1,249	115
Mali	2,354	81
Mauritania	4,798	164

Mauritius	24,967	1,207
Mozambique	1,339	62
Namibia	12,024	969
Niger	1,271	61
Nigeria	6,130	214
Rwanda	2,397	130
São Tomé and Príncipe	3,506	197
Senegal	3,893	144
Seychelles	31,889	1,123
Sierra Leone	1,707	244
South Africa	14,042	1,071
Eswatini	10,477	663
Tanzania	3,679	112
Togo	1,818	100
Uganda	2,618	117
Zambia	4,264	175
Zimbabwe	2,470	185
South Sudan	1,443	148
Djibouti	4,016	122
Egypt	14,081	516
Libya	12,100	627
Morocco	9,339	466
Somalia	1,467	15
Sudan	4,109	298
Tunisia	12,863	806
